# Prognosis of pulmonary fibrosis presenting with a usual interstitial pneumonia pattern on computed tomography in patients with myeloperoxidase anti-neutrophil cytoplasmic antibody-related nephritis: a retrospective single-center study

**DOI:** 10.1186/s12890-019-0969-5

**Published:** 2019-11-01

**Authors:** Toshikazu Watanabe, Tomoyuki Minezawa, Midori Hasegawa, Yasuhiro Goto, Takuya Okamura, Yosuke Sakakibara, Yoshikazu Niwa, Atsushi Kato, Masamichi Hayashi, Sumito Isogai, Masashi Kondo, Naoki Yamamoto, Naozumi Hashimoto, Kazuyoshi Imaizumi

**Affiliations:** 1 Department of Respiratory Medicine, Fujita Health University School of Medicine, 1-98 Dengakugakubo, Kutsukake-cho, Toyoake, Aichi 470-1192 Japan; 2 Department of Nephrology, Fujita Health University School of Medicine, 1-98 Dengakugakubo, Kutsukake-cho, Toyoake, Aichi 470-1192 Japan; 3 Center for Joint Research Facilities Support , Research Promotion and Support Headquarters, Fujita Health University School of Medicine, 1-98 Dengakugakubo, Kutsukake-cho, Toyoake, Aichi 470-1192 Japan; 4 Department of Respiratory Medicine, Nagoya University Graduate School of Medicine, 65 Tsurumai-cho, Showa-ku, Nagoya, 466-8550 Japan

**Keywords:** Myeloperoxidase anti-neutrophil cytoplasmic antibody-related nephritis, Pulmonary fibrosis, Three-dimensional computed tomography, Usual interstitial pneumonia, Idiopathic pulmonary fibrosis

## Abstract

**Background:**

Myeloperoxidase anti-neutrophil cytoplasmic antibody-related nephritis (MPO-ANCA nephritis) is occasionally accompanied by lung abnormalities such as pulmonary fibrosis. However, the clinical features of pulmonary fibrosis in patients with MPO-ANCA nephritis have not been well documented. This study was performed to compare the prognosis of a usual interstitial pneumonia (UIP) pattern of lung fibrosis in patients with MPO-ANCA nephritis with the prognosis of idiopathic pulmonary fibrosis (IPF).

**Methods:**

We retrospectively reviewed the medical records of 126 patients with MPO-ANCA nephritis and identified 31 with a UIP pattern of lung fibrosis on high-resolution or thin-slice computed tomography (CT). We compared the characteristics and prognosis of these patients with those of 32 patients with IPF. In 18 patients from both groups, we assessed and compared the decline in lung volume over time using three-dimensional (3D) CT images reconstructed from thin-section CT data.

**Results:**

The numbers of male and female patients were nearly equal among patients with MPO-ANCA nephritis exhibiting a UIP pattern; in contrast, significant male dominancy was observed among patients with IPF (*p* = 0.0021). Significantly fewer smokers were present among the patients with MPO-ANCA nephritis with a UIP pattern than among those with IPF (*p* = 0.0062). There was no significant difference in the median survival time between patients with MPO-ANCA nephritis with a UIP pattern (50.8 months) and IPF (55.8 months; *p* = 0.65). All patients with IPF in this cohort received antifibrotic therapy (pirfenidone or nintedanib). Almost half of the deaths that occurred in patients with MPO-ANCA nephritis with a UIP pattern were caused by non-respiratory-related events, whereas most deaths in patients with IPF were caused by respiratory failure such as acute exacerbation. In the 3D CT lung volume analyses, the rate of decline in lung volume was equivalent in both groups.

**Conclusions:**

MPO-ANCA nephritis with a UIP pattern on CT may have an unfavorable prognosis equivalent to that of IPF with a UIP pattern treated with antifibrotic agents.

## Background

Anti-neutrophil cytoplasmic antibody (ANCA)-associated vasculitis (AAV) includes three main types of diseases: microscopic polyangiitis (MPA), granulomatous polyangiitis (GPA), and eosinophilic granulomatous polyangiitis. GPA and MPA are frequently accompanied by glomerulonephritis within 2 years after the onset of vasculitis [1, 2]. Pulmonary fibrosis develops less frequently than glomerulonephritis in patients with AAV. Among the three types of AAV, MPA, which is strongly associated with myeloperoxidase ANCA (MPO-ANCA), is most frequently accompanied by pulmonary fibrosis (15–47% of cases) [3–6]. MPO-ANCA-associated vasculitis more frequently occurs in Japanese patients, whereas proteinase-3 ANCA-associated vasculitis is more common in Europe and the United States [7]. Because both glomerulonephritis and pulmonary fibrosis are known to be unfavorable prognostic factors in patients with AAV, management of pulmonary fibrosis in patients with MPO-ANCA-related nephritis (MPO-ANCA nephritis) is a critical clinical problem [8, 9]. In the real-world setting, nephrologists often consult with pulmonologists about the management of pulmonary fibrosis in patients with MPO-ANCA nephritis. Some previous studies have addressed the relationship between MPO-ANCA and pulmonary fibrosis, showing that MPO-ANCA positivity is associated with a poor prognosis of pulmonary fibrosis [10]. However, because these studies focused mainly on pulmonary fibrosis, they may have included patients whose vasculitis activities were indolent or silent. Thus, the detailed clinical features of pulmonary fibrosis in patients with overt ANCA-related pathological conditions (i.e., those affecting other organs such as the kidney) have not been fully elucidated.

Previous studies have shown that a usual interstitial pneumonia (UIP) pattern is the most prevalent pattern on high-resolution computed tomography (HRCT) in patients with MPO-ANCA-associated interstitial pneumonia [11]. A UIP pattern on HRCT is a distinct CT finding characterized by honeycombing, reticular shadows, and traction bronchiectasis [12]. Idiopathic pulmonary fibrosis (IPF), which typically exhibits the UIP pattern on HRCT as well as the pathological pattern of UIP, has a significantly worse prognosis than chronic fibrotic idiopathic interstitial pneumonia [12, 13]. The aim of this study was to compare the clinical features and prognosis of pulmonary fibrosis showing a UIP pattern in patients with MPO-ANCA nephritis versus IPF.

## Patients and methods

### Patients

We retrospectively reviewed the records of 126 consecutive patients with MPO-ANCA nephritis who visited the Department of Nephrology in Fujita Health University Hospital from 2008 to 2018. Among these patients, we identified 31 patients who had lung fibrosis with a UIP pattern on thin-section CT (TSCT) or HRCT. All 31 patients had visited the Department of Respiratory Medicine for consultation within 1 month after the nephrologist first noted co-existing lung fibrosis. We simultaneously selected 32 patients with IPF who had been diagnosed and treated at Fujita Health University Hospital from 2011 to 2018. The diagnosis of IPF or a UIP pattern on CT was based on the criteria in the clinical practice guideline of the American Thoracic Society/European Respiratory Society/Japanese Respiratory Society/Latin American Thoracic Association [13]. The patients with IPF comprised 9 patients with a pathologically proven diagnosis obtained by thoracoscopic biopsy and 23 patients diagnosed by typical HRCT findings (UIP pattern) along with other consistent clinical findings. We excluded patients with serum ANCA positivity from among the patients with IPF. All patients with IPF had received antifibrotic therapy with either pirfenidone or nintedanib for at least 6 months. Overall survival was defined as the time from the first hospital visit (in IPF patients) or initial consultation with the pulmonologist for co-existing lung fibrosis (in ANCA-nephritis patients) to the date of death or last follow-up visit.

### Evaluation of HRCT findings

**Fig. 1:**
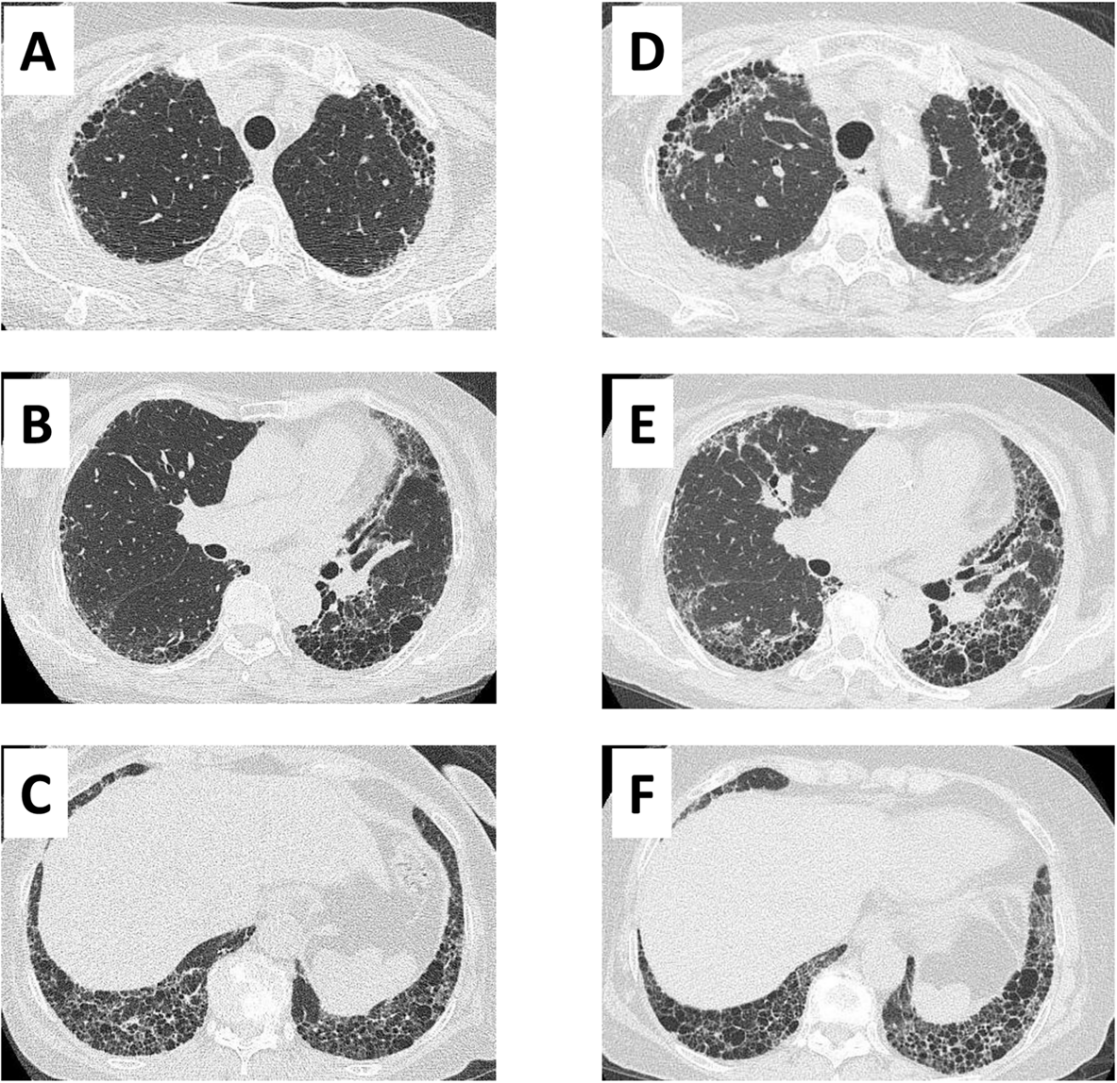
Representative thin-slice CT images of the lung from a patient with ANCA nephritis with a UIP pattern. (**a**–**c**) CT images at initial examination. (**d**–**f**) CT images 48 months after the initial CT examination. CT, computed tomography; ANCA, anti-neutrophil cytoplasmic antibody; UIP, usual interstitial pneumonia

**Fig. 2:**
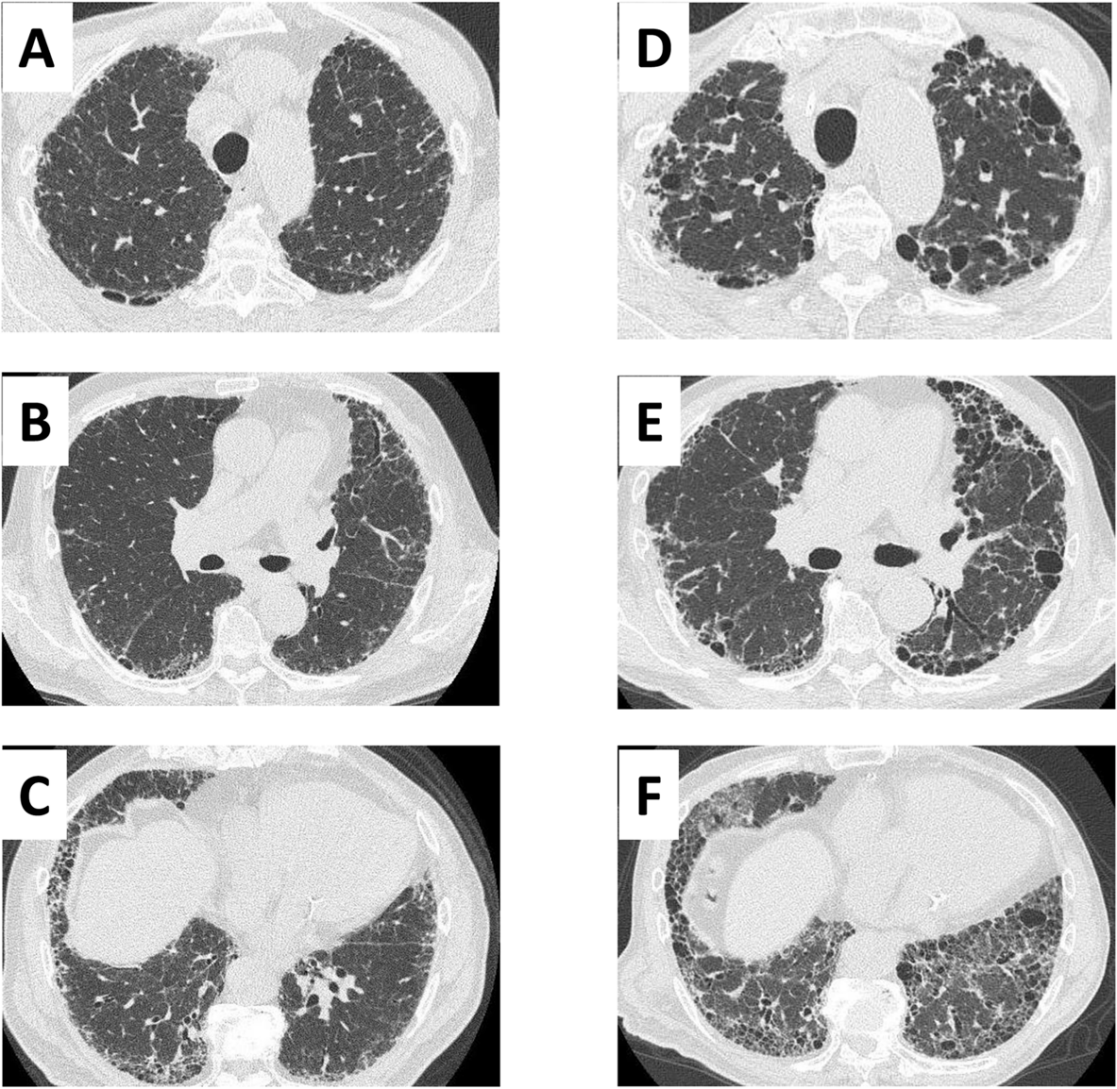
Representative thin-slice CT images of the lung from a patient with IPF. (**a**–**c**) CT images at initial examination. (**d**–**f**) CT images 36 months after the initial CT examination. CT, computed tomography; IPF, idiopathic pulmonary fibrosis

CT (0.5 mm slices) images were generated using a non-enhanced multidetector CT system (Aquilion One; Toshiba Medical Systems, Tokyo, Japan). The CT scan parameters were as follows: tube current, automatic exposure control SD7; tube voltage, 120 kV; rotation speed, 0.5 s/rot; table speed, BP 1.39; reconstruction filter, FC51 AIDR 3D WEAK; window width, 1600; and window level, − 600. We used automatic exposure control, the volume helical scan mode, and multi-planar reconstruction images. Three observers, including two experts in thoracic radiology for diffuse lung diseases (T.W. and T.M. with 5 and 10 years’ experience of image diagnosis of pulmonary fibrosis, respectively) and one respiratory medicine specialist (K.I.) independently evaluated the TSCT or HRCT images of each patient within 3 months after the first visit to the hospital. The three observers selected one of the four categories of CT patterns: UIP or probable UIP pattern (defined as UIP pattern in this study), indeterminate for UIP pattern, alternative diagnosis pattern, and no abnormal change [13]. When three observers’ conclusions did not coincide, the final decision was made by group discussion. The kappa coefficient was calculated for evaluation of interobserver agreement. Representative UIP patterns in CT images from patients with ANCA-nephritis and IPF at two time points are shown in Figs. 1 and 2, respectively.

### Evaluation of lung volume by three-dimensional CT

**Fig. 3:**
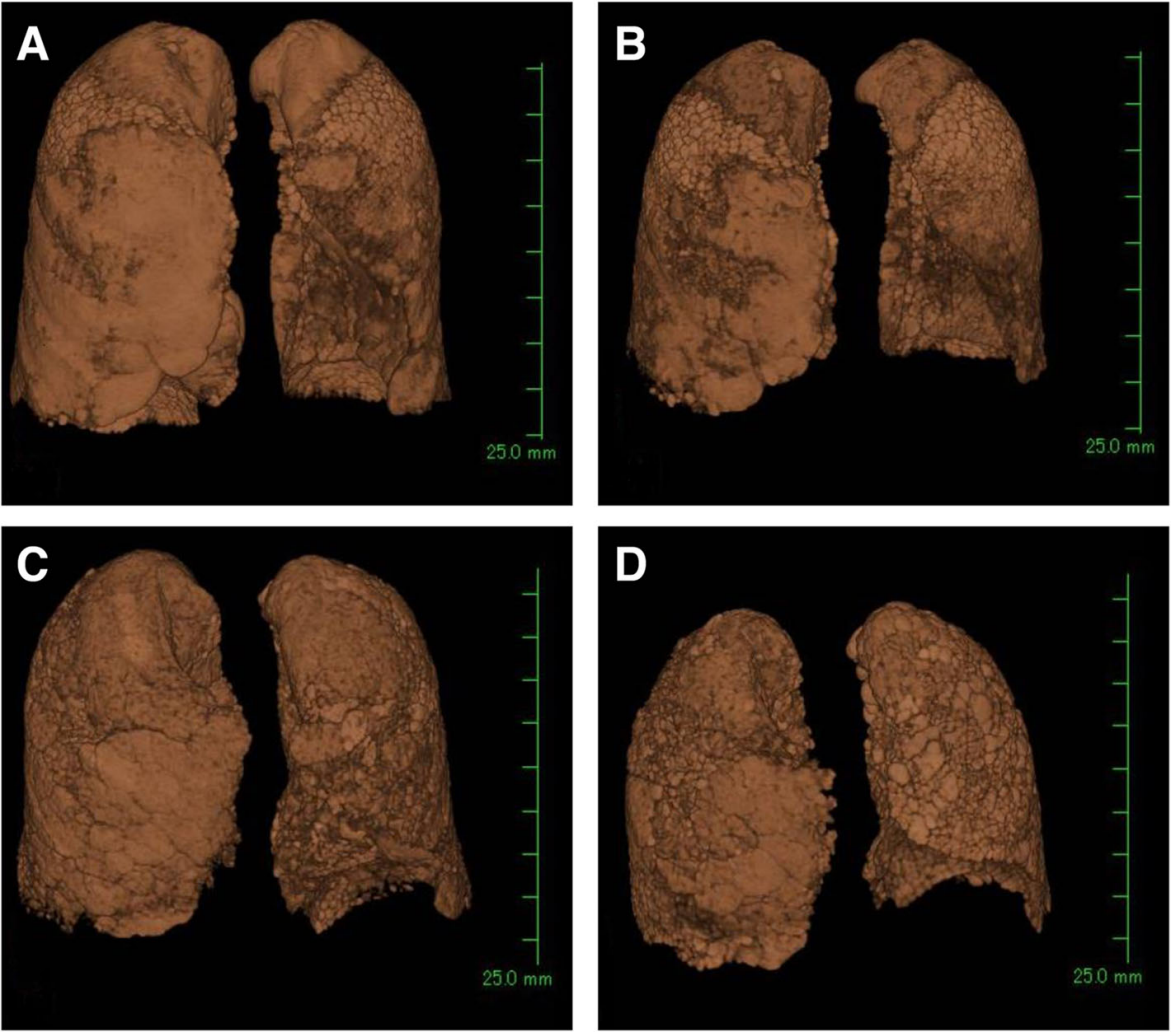
Representative data of three-dimensional reconstruction images of the lung from the CT data at the initial and following examinations. (**a**, **b**) ANCA nephritis with a UIP pattern. (**c**, **d**) IPF. (**a**, **c**) Whole-lung images reconstructed from the data of the initial CT examination. (**b**) CT image 48 months after the initial CT examination. (**d**) CT image 36 months after the initial CT examination. CT, computed tomography; ANCA, anti-neutrophil cytoplasmic antibody; UIP, usual interstitial pneumonia; IPF, idiopathic pulmonary fibrosis

Eighteen patients were selected from both groups of patients (those with MPO-ANCA nephritis with a UIP pattern and those with IPF) who had undergone TSCT (Aquilion ONE ViSION; Toshiba Medical Systems, Tokyo, Japan) at least twice, including the first time when physicians recognized pulmonary fibrosis and a second or later time point at least 6 months after the first TSCT. We reconstructed three-dimensional (3D) images of the whole lung from these TSCT data and calculated the lung volume using 3D reconstruction software (AZE Virtual Place; AZE Ltd., Tokyo, Japan) (Fig. 3). In brief, a 3D image of the lung was generated by extracting lung and bronchial images from the TSCT data followed by subtraction of the bronchial fraction. The software automatically calculated the volume of the created lungs. Next, we analyzed the difference (decline) in the 3D image lung volume between the first and latest TSCT. The annual change was calculated assuming that the lung volume declined at a regular pace. We evaluated the net and percent lung volume decline from baseline. We did not use the CT data just before death (within 1 month) because the lung volume might have been affected by various serious end-stage conditions such as pneumothorax, diffuse alveolar damage, or infection. In addition, nine patients with IPF who had undergone video-assisted thoracoscopic surgical biopsy were excluded because the surgical procedure could have affected expansion of the lung.

### Statistical analysis

We used the nonparametric Wilcoxon signed-rank test for comparative analyses between the two groups. The kappa coefficient was used to assess interobserver agreement in the CT evaluation. Survival curves were evaluated by the Kaplan–Meier method, and the median survival time was analyzed by the log-rank test. All statistical analyses were performed using JMP version 13 (SAS Institute, Tokyo, Japan).

## Results

### CT patterns in patients with MPO-ANCA nephritis

The chest CT patterns in patients with MPO-ANCA nephritis are shown in Table 1. Among 126 patients with MPO-ANCA nephritis, 31 (24.6%) had a UIP or probable UIP pattern, 8 (6.3%) had findings indeterminate for a UIP pattern, and 27 (21.4%) had pulmonary infiltrations suggestive of a diagnosis other than UIP (alternative diagnosis). Sixty patients (47.6%) had no pulmonary infiltration. The Interobserver agreement as evaluated by the kappa coefficient was good or excellent (range, 0.79–0.97).

**Table 1:** Chest CT findings in patients with ANCA nephritis (*n* = 126) and concordance among three observers

CT pattern	Observer 1	Observer 2	Observer 3	After discussion
UIP or probable UIP	20	31	30	31
Indeterminate for UIP	20	6	12	8
Alternative diagnosis	30	29	24	27
No abnormal findings	56	60	60	60
Kappa coefficient				
Observers 1/2	0.792			
Observers 1/3	0.817			
Observers 2/3	0.975			

Observers 1 and 2: specialists in thoracic radiology;

Observer 3: specialist in respiratory medicine

*CT* computed tomography, *ANCA* anti-neutrophil cytoplasmic antibody, *UIP* usual interstitial pneumonia

### Patient characteristics

The clinical features of the 31 patients with MPO-ANCA nephritis with a UIP pattern and the 32 patients with IPF retrospectively recruited in this study are summarized in Table 2. The clinical backgrounds, respiratory symptoms, lactate dehydrogenase level, and Krebs von den Lungen-6 glycoprotein level were equivalent in both groups. Significantly more male patients and smokers (current and ex-smokers) were present among the patients with IPF (*p* = 0.0021 and *p* = 0.0062, respectively). Table 3 shows the details of renal dysfunction in the patients with MPO-ANCA nephritis with a UIP pattern. All but one patient had microscopic polyangiitis. The mean estimated glomerular filtration rate and serum creatinine level in the patients with MPO-ANCA nephritis with a UIP pattern were 34.1 mL/min/1.73 m^2^ and 2.66 mg/dL, respectively. Eight patients with MPO-ANCA nephritis with a UIP pattern had undergone hemodialysis, whereas no patients with IPF had started dialysis. Most patients with MPO-ANCA nephritis with a UIP pattern had been treated with immunosuppressive agents (*n* = 28, 90.0%), although two patients (6.4%) had received no treatment. Among the 28 patients with ANCA-nephritis who had received immunosuppressive therapy, seven (22.5%) experienced relapses after transient improvements of nephritis. Most recurrences occurred during tapering of the corticosteroid dose, and all relapsed patients subsequently recovered after re-increasing the steroid dose. All patients with IPF had been treated with antifibrotic agents (nintedanib *n* = 14 (300 mg/day *n* = 4, 200 mg/day *n* = 10) or pirfenidone *n* = 18 (1800 mg/day *n* = 3, 1200 mg/day *n* = 11, 600 mg/day n = 4)), whereas only one patient with MPO-ANCA nephritis had received antifibrotic therapy (nintedanib 300 mg/day) (Table 2).

**Table 2:** Clinical characteristics of patients with MPO-ANCA nephritis with a UIP pattern and patients with IPF

Characteristics	MPO-ANCA nephritis with UIP pattern	IPF	*p*-value
Patients	31	32	
Age, years	74 (58–88)	70 (58–87)	0.083
Male	16	29	0.0021
Smoking history			
Never/former or current	16/15	6/26	0.0068
Respiratory symptoms			
Cough	19	16	0.37
Dyspnea	12	18	0.16
None	9	6	0.34
Serological testing			
LDH, U/L	225.5 ± 64.9	236.2 ± 45.1	0.35
KL-6, IU/mL	1298 ± 914.8	1341 ± 853.5	0.73
Treatment			
None	2 (6.5)	0 (0.0)	
Immunosuppressive agents	28 (90.0)	0 (0.0)	
Antifibrotic agents	1 (3.2)	32 (100.0)	
	nintedanib	nintedanib n = 14	
	300 mg/day	300 mg/day n = 4	
		200 mg/day n = 10	
		pirfenidone n = 18	
		1800 mg/day n = 3	
		1200 mg/day n = 11	
		600 mg/day n = 4	

Data are presented as n, median (range), mean ± standard deviation, or n (%)

*MPO-ANCA nephritis* myeloperoxidase anti-neutrophil cytoplasmic antibody-related nephritis, *UIP* usual interstitial pneumonia, *IPF* idiopathic pulmonary fibrosis, *LDH* lactate dehydrogenase, *KL-6* Krebs von den Lungen-6

**Table 3:** Renal findings in patients with MPO-ANCA nephritis with a UIP pattern

MPO-ANCA nephritis with UIP pattern (*n* = 31)	
Disease phenotype	
MPA / GPA	30 / 1
Renal function	
eGFR, mL/min/1.73 m^2^	34.1 ± 24.3
Serum creatinine, mg/dL	2.66 ± 2.41
Urinalysis findings	
Semi-quantitative urinary protein, − / 1+ / 2+ / 3+	13 / 5 / 10 / 3
Urinary occult blood, − / 1+ / 2+ / 3+	2 / 8 / 3 / 18
Urinary protein/creatinine ratio, < 0.5 / ≥0.5 g/gCr	15 / 16
Undergoing dialysis	8

Data are presented as number of patients or mean ± standard deviation

*MPO-ANCA nephritis* myeloperoxidase anti-neutrophil cytoplasmic antibody-related nephritis, *MPA* microscopic polyangiitis, *GPA* granulomatosis with polyangiitis, *UIP* usual interstitial pneumonia, *eGFR* estimated glomerular filtration rate

### Prognostic analysis of patients with MPO-ANCA nephritis with a UIP pattern

**Fig. 4:**
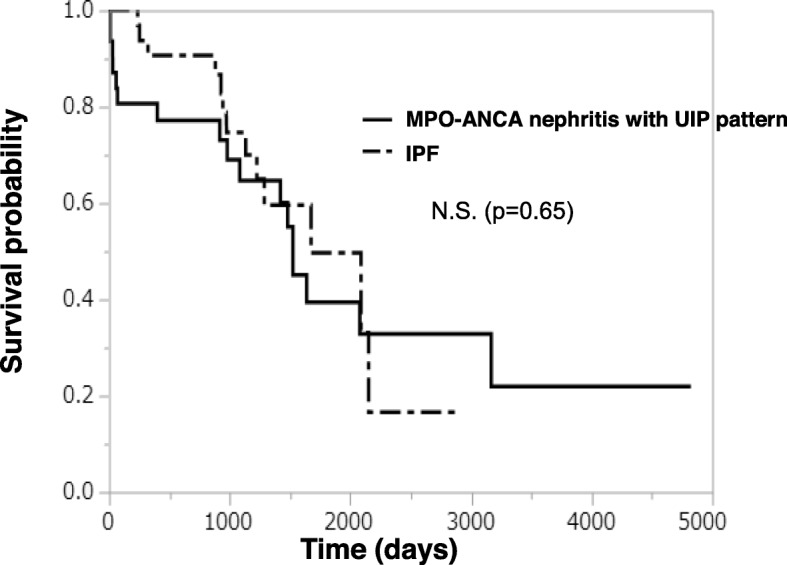
Kaplan–Meier distribution of survival rates in patients with MPO-ANCA nephritis with a UIP pattern versus those with IPF. MPO-ANCA, myeloperoxidase anti-neutrophil cytoplasmic antibody-related nephritis; UIP, usual interstitial pneumonia; N.S., no significant difference

**Fig. 5:**
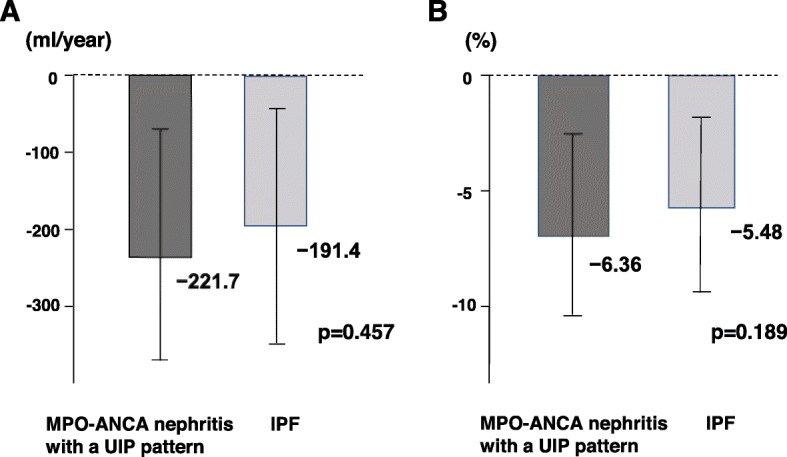
Comparison of annual decline rate of lung volume from baseline calculated from three-dimensional computed tomography data between patients with MPO-ANCA nephritis with a UIP pattern and IPF. (**a**) Net lung volume decline and (**b**) percent lung volume decline. MPO-ANCA, myeloperoxidase anti-neutrophil cytoplasmic antibody-related nephritis; UIP, usual interstitial pneumonia; IPF, idiopathic pulmonary fibrosis

The results of the survival analysis between the patients with MPO-ANCA nephritis with a UIP pattern and those with IPF are shown in Fig. 4. The median survival time of the patients with MPO-ANCA nephritis and IPF was 50.8 and 55.8 months, respectively, with no significant difference (*p* = 0.65). We also analyzed the annual decline in lung volume calculated by 3D images of the lung reconstructed from TSCT data. The net annual decline in lung volume was − 221.7 mL/year in the patients with MPO-ANCA nephritis with a UIP pattern and − 191.4 mL/year in those with IPF (Fig. 5a); the difference was not statistically significant (*p* = 0.457). Similarly, the annual percent change from the baseline lung volume was − 6.36% per year in the patients with MPO-ANCA nephritis with a UIP pattern and − 5.48% in those with IPF (Fig. 5b), again with no significant difference (*p* = 0.189).

### Causes of death

In total, 17 patients with MPO-ANCA nephritis with a UIP pattern and 13 patients with IPF died during the follow-up period. Causes of death in both groups are summarized in Table 4. There was an obvious difference in the causes of death between the two groups. Among the patients with MPO-ANCA nephritis with a UIP pattern, about half of the deceased patients (*n* = 8) had died of non-respiratory causes. In contrast, among the patients with IPF, 92% of the deceased patients (*n* = 12) had died of respiratory causes. Acute exacerbation (38%) and respiratory failure (38%) were the two major causes of death in patients with IPF. Notably, four patients with MPO-ANCA nephritis with a UIP pattern but none with IPF had died of alveolar hemorrhage.

**Table 4:** Causes of death in patients with MPO-ANCA nephritis with a UIP pattern and patients with IPF

	MPO-ANCA nephritis with UIP pattern (*n* = 31)	IPF (*n* = 32)
Number of deaths	17	13
Cause of death		
Respiratory-related death	9 (53)	12 (92)
Acute exacerbation	2	5
Chronic respiratory failure	1	5
Pneumonia	1	0
Pneumothorax	1	2
Alveolar hemorrhage	4	0
Non-respiratory-related death	8 (47)	1 (8)
Sepsis	3	0
Cardiovascular disease	4	1
Others	1^a^	0

Data are presented as n or n (%)

^a^Ovarian cancer

*MPO-ANCA nephritis* myeloperoxidase anti-neutrophil cytoplasmic antibody-related nephritis, *UIP* usual interstitial pneumonia, *IPF* idiopathic pulmonary fibrosis

## Discussion

Some excellent studies have addressed the clinical features, including the prognosis of pulmonary fibrosis, in patients with serum MPO-ANCA positivity [10, 14]. These studies showed that the prognosis of MPO-ANCA-positive pulmonary fibrosis was worse than that of ANCA-negative pulmonary fibrosis associated with other collagen vascular diseases. However, few studies have focused on the development of pulmonary fibrosis in patients with MPO-ANCA nephritis. A previous review showed that pulmonary fibrosis in patients with MPA and GPA exhibits various HRCT patterns, including the typical UIP pattern with honeycombing in the basal lung (most common, 47%), a combined pulmonary fibrosis and emphysema pattern, and a fibrotic nonspecific interstitial pneumonia pattern [15, 16]. Hosoda et al. [17] reported that the clinical features of MPO-ANCA-positive UIP without any overt collagen diseases were distinguishable from the clinical features of IPF. Thus, the present study, which is the first study to elucidate the prognosis of a UIP pattern of pulmonary fibrosis in patients with MPO-ANCA nephritis, may have clinical relevance.

Tzelepis et al. [18] reported that the overall survival of patients with MPO-ANCA nephritis with pulmonary fibrosis was 72 months, which is more favorable than in the present study. However, their study included pulmonary fibrosis with various chest CT patterns, not only a UIP pattern. In addition, they included younger patients than in our study. Conversely, the median survival time of patients with IPF in the present study was 55.8 months, which is more favorable than previously reported in Japan [19] and Western countries [20–23]. Notably, our patients with IPF had been treated with antifibrotic agents (nintedanib or pirfenidone). These previous articles were published before these antifibrotic agents had been introduced to daily clinical practice. Although there is no clear evidence that antifibrotic agents improve the survival of patients with IPF, some research has shown that pirfenidone might reduce mortality and improve life expectancy compared with best supportive care [24, 25]. We speculate that our patients with IPF might have had a more favorable prognosis because they had all received either pirfenidone or nintedanib. Our study showed that MPO-ANCA nephritis with a UIP pattern might have a poor prognosis similar to that of IPF under the appropriate therapy for each type of disease (anti-inflammatory therapy for MPO-ANCA nephritis and antifibrotic therapy for IPF).

Although our study showed no significant difference in prognosis, we found a striking difference in the causes of death between MPO-ANCA nephritis with a UIP pattern and IPF. Among patients with IPF, 92% of deaths were respiratory-related. These results are consistent with those of an epidemiologic study of Japanese patients with IPF [19]. In contrast, in patients with MPO-ANCA nephritis with a UIP pattern, deaths were more frequently related to anti-immune therapy (infectious complication) or vasculitis itself (alveolar hemorrhage and perhaps cardiovascular disease). Intriguingly, though the decline in lung volume and median survival time were similar between the two groups in this study, the causes of death were different. This might indicate that the respiratory impairment in patients with IPF may not be caused only by low vital capacity; other factors (impairment of diffusion capacity or acute exacerbation) may also have a significant prognostic influence.

In this study, we used 3D CT reconstruction data of the whole lung to evaluate the annual decline in lung volume. Because this study was a retrospective analysis of data from patients with nephritis (most patients were followed up mainly in the nephrology department), spirometry data obtained through regular follow-up were not available. Iwano et al. [26] reported that lung volume calculated using 3D CT volumetry was well correlated with lung volume measured using spirometry. Because the decline of vital capacity in spirometry is an important prognostic factor in patients with IPF, we substituted the decline in lung volume calculated from 3D CT for the spirometric decline in vital capacity. Although further studies are needed to confirm the reliability, we believe that lung volume data calculated from CT images could be useful for evaluation of pulmonary restrictive impairment.

This study has some limitations. First, this was a single-center retrospective study involving a small number of patients. Second, we were unable to analyze pulmonary function test data. Thus, the decline in the true vital capacity and diffusion capacity could not be evaluated in this study. In addition, we cannot rule out the possibility that the decline in vital capacity calculated from reconstructed 3D lung images might have been influenced by timing or the time interval in which chest CT was performed. Finally, we selected patients with MPO-ANCA nephritis with a UIP pattern only by HRCT findings, not pathological findings. In addition, we did not include patients in whom pulmonary fibrosis preceded the onset of nephritis. The time interval between the nephritis diagnosis and initial consultation with the pulmonologist depended on the individual nephrologist, but the mean interval between diagnosis of nephritis and diagnosis of UIP (lung fibrosis) was 18 months. The patients with ANCA-nephritis UIP in our study might thus have had relatively advanced disease and we might have excluded more patients with mild or early-stage fibrosis with a UIP pattern from the patients with ANCA nephritis. Further multicenter prospective studies with large numbers of patients are needed to fully elucidate the clinical characteristics of MPO-ANCA nephritis with a UIP pattern.

## Conclusions

The prognosis of MPO-ANCA nephritis with a UIP pattern is poor and equivalent to that of IPF treated with antifibrotic agents. The lung volume reduction rate might be equivalent in both diseases. However, according to the present study, non-respiratory events are the cause of death in nearly half of patients with MPO-ANCA nephritis with a UIP pattern, whereas respiratory events are the cause of death in most patients with IPF.

## Data Availability

The datasets used and/or analyzed during the current study are available from the corresponding author on reasonable request.

## Abbreviations

3D: Three-dimensional
AAV: Anti-neutrophil cytoplasmic antibody-associated vasculitis
ANCA: Anti-neutrophil cytoplasmic antibody
CT: Computed tomography
GPA: Granulomatous polyangiitis
HRCT: High-resolution computed tomography
IPF: Idiopathic pulmonary fibrosis
MPA: Microscopic polyangiitis
MPO: Myeloperoxidase
TSCT: Thin-section computed tomography
UIP: Usual interstitial pneumonia

## References

[1] Agard C, Mouthon L, Mahr A, Guillevin L (2003). Microscopic polyangiitis and polyarteritis nodosa: how and when do they start?. Arthritis Rheum.

[2] Guillevin L, Durand-Gasselin B, Cevallos R, Gayraud M, Lhote F, Callard P (1999). Microscopic polyangiitis: clinical and laboratory findings in eighty-five patients. Arthritis Rheum.

[3] Sada KE, Yamamura M, Harigai M, Fujii T, Dobashi H, Takasaki Y (2014). Classification and characteristics of Japanese patients with antineutrophil cytoplasmic antibody-associated vasculitis in a nationwide, prospective, inception cohort study. Arthritis Res Ther.

[4] Schirmer JH, Wright MN, Vonthein R, Herrmann K, Nolle B, Both M (2016). Clinical presentation and long-term outcome of 144 patients with microscopic polyangiitis in a monocentric German cohort. Rheumatology (Oxford).

[5] Guillevin L, Lhote F (1995). Distinguishing polyarteritis nodosa from microscopic polyangiitis and implications for treatment. Curr Opin Rheumatol.

[6] Lane SE, Watts RA, Shepstone L, Scott DG (2005). Primary systemic vasculitis: clinical features and mortality. QJM..

[7] Ozaki S (2007). ANCA-associated vasculitis: diagnostic and therapeutic strategy. Allergol Int.

[8] Mukhtyar C, Flossmann O, Hellmich B, Bacon P, Cid M, Cohen-Tervaert JW (2008). Outcomes from studies of antineutrophil cytoplasm antibody associated vasculitis: a systematic review by the European league against rheumatism systemic vasculitis task force. Ann Rheum Dis.

[9] Hirayama K, Kobayashi M, Usui J, Arimura Y, Sugiyama H, Nitta K (2015). Pulmonary involvements of anti-neutrophil cytoplasmic autoantibody-associated renal vasculitis in Japan. Nephrol Dial Transplant.

[10] Homma S, Suzuki A, Sato K (2013). Pulmonary involvement in ANCA-associated vasculitis from the view of the pulmonologist. Clin Exp Nephrol.

[11] Mohammad AJ, Mortensen KH, Babar J, Smith R, Jones RB, Nakagomi D (2017). Pulmonary involvement in antineutrophil cytoplasmic antibodies (ANCA)-associated vasculitis: the influence of ANCA subtype. J Rheumatol.

[12] Lynch DA, Sverzellati N, Travis WD, Colby TV, Inoue Y, Nicholson AG (2018). Diagnostic criteria for idiopathic pulmonary fibrosis: a Fleischner society white paper. Lancet Respir Med.

[13] Raghu G, Remin-Jardin M, Myers JL, Richeldy L, Ryercon CJ, Lederer DJ (2018). Diagnosis of idiopathic pulmonary fibrosis: an official ATS/ERS/JRS/ALAT clinical practice guidelines. Am J Respir Crit Care Med.

[14] Homma S, Matsushita H, Nakata K (2014). Pulmonary fibrosis in myeloperoxidase antineutrophil cytoplasmic antibody-associated vasculitides. Respirology..

[15] Chen M, Yu F, Zhang Y, Zhao MH (2008). Antineutrophil cytoplasmic autoantibody-associated vasculitis in older patients. Medicine (Baltimore).

[16] Comarmond C, Crestani B, Tazi A, Hervier B, Adam-Marchand S, Nunes H (2014). Pulmonary fibrosis in antineutrophil cytoplasmic antibodies (ANCA)-associated vasculitis: a series of 49 patients and review of the literature. Medicine (Baltimore).

[17] Hosoda C, Baba T, Hagiwara E, Ito H, Matsuo N, Kitamura H (2016). Clinical features of usual interstitial pneumonia with anti-neutrophil cytoplasmic antibody in comparison with idiopathic pulmonary fibrosis. Respirology..

[18] Tzelepis GE, Kokosi M, Tzioufas A, Toya SP, Boki KA, Zormpala A (2010). Prevalence and outcome of pulmonary fibrosis in microscopic polyangiitis. Eur Respir J.

[19] Natsuizaka M, Chiba H, Kuronuma K, Otsuka M, Kudo K, Mori M (2014). Epidemiologic survey of Japanese patients with idiopathic pulmonary fibrosis and investigation of ethnic differences. Am J Respir Crit Care Med.

[20] Bjoraker JA, Ryu JH, Edwin MK, Myers JL, Tazelaar HD, Schroeder DR (1998). Prognostic significance of histopathologic subsets in idiopathic pulmonary fibrosis. Am J Respir Crit Care Med.

[21] King TE, Tooze JA, Schwarz MI, Brown KR, Cherniack RM (2001). Predicting survival in idiopathic pulmonary fibrosis: scoring system and survival model. Am J Respir Crit Care Med.

[22] Mapel DW, Hunt WC, Utton R, Baumgartner KB, Samet JM, Coultas DB (1998). Idiopathic pulmonary fibrosis: survival in population based and hospital based cohorts. Thorax..

[23] Schwartz DA, Helmers RA, Galvin JR, Van Fossen DS, Frees KL, Dayton CS (1994). Determinants of survival in idiopathic pulmonary fibrosis. Am J Respir Crit Care Med.

[24] Fisher M, Nathan SD, Hill C, Marshall J, Dejonckheere F, Thuresson PO (2017). Predicting life expectancy for pirfenidone in idiopathic pulmonary fibrosis. J Manag Care Spec Pharm.

[25] Nathan SD, Albera C, Bradford WZ, Costabel U, Glaspole I, Glassberg MK (2017). Effect of pirfenidone on mortality: pooled analyses and meta-analyses of clinical trials in idiopathic pulmonary fibrosis. Lancet Respir Med.

[26] Iwano S, Okada T, Satake H, Naganawa S (2009). 3D-CT volumetry of the lung using multidetector row CT: comparison with pulmonary function tests. Acad Radiol.

